# Diagnostic yield of next-generation sequencing in 87 families with neurodevelopmental disorders

**DOI:** 10.1186/s13023-022-02213-z

**Published:** 2022-02-19

**Authors:** María Isabel Álvarez-Mora, Aurora Sánchez, Laia Rodríguez-Revenga, Jordi Corominas, Raquel Rabionet, Susana Puig, Irene Madrigal

**Affiliations:** 1 Biochemistry and Molecular Genetics Department, Hospital Clinic of Barcelona, IDIBAPS (Institut de Investigacions Biomèdiques August Pi I Sunyer), Barcelona, Spain; 2 CIBER of Rare Diseases (CIBERER), Barcelona, Spain; 3 Department of Human Genetics, Radboud UMC, Nijmegen, The Netherlands; 4 Department of Genetics, Microbiology and Statistics, Faculty of Biology, University of Barcelona, Barcelona, Spain; 5 Department of Dermatology, Hospital Clínic of Barcelona and Melanoma Unit, IDIBAPS, Barcelona, Spain

**Keywords:** Neurodevelopmental disorders, Whole exome sequencing, Whole genome sequencing, Genetic diagnostic, Genetic diagnostic yield

## Abstract

**Background:**

Neurodevelopmental disorders (NDDs) are a group of heterogeneous conditions, which include mainly intellectual disability, developmental delay (DD) and autism spectrum disorder (ASD), among others. These diseases are highly heterogeneous and both genetic and environmental factors play an important role in many of them. The introduction of next generation sequencing (NGS) has lead to the detection of genetic variants in several genetic diseases. The main aim of this report is to discuss the impact and advantages of the implementation of NGS in the diagnosis of NDDs. Herein, we report diagnostic yields of applying whole exome sequencing in 87 families affected by NDDs and additional data of whole genome sequencing (WGS) from 12 of these families.

**Results:**

The use of NGS technologies allowed identifying the causative gene alteration in approximately 36% (31/87) of the families. Among them, de novo mutation represented the most common cause of genetic alteration found in 48% (15/31) of the patients with diagnostic mutations. The majority of variants were located in known neurodevelopmental disorders genes. Nevertheless, some of the diagnoses were made after the use of GeneMatcher tools which allow the identification of additional patients carrying mutations in *THOC2*, *SETD1B* and *CHD9* genes. Finally the use of WGS only allowed the identification of disease causing variants in 8% (1/12) of the patients in which previous WES failed to identify a genetic aetiology.

**Conclusion:**

NGS is more powerful in identifying causative pathogenic variant than conventional algorithms based on chromosomal microarray as first-tier test. Our results reinforce the implementation of NGS as a first-test in genetic diagnosis of NDDs.

## Background

Neurodevelopmental disorders (NDDs) are a group of heterogeneous conditions that result from an abnormal brain development that may give rise to impaired cognition, communication, adaptive behaviour, and psychomotor skills [1]. NDDs include several disorders such as autism spectrum disorder (ASD), social communication disorders, intellectual disability (ID), developmental delay (DD) or attention deficit hyperactivity disorder (ADHD), among others. NDDs constitute a serious health problem in our society, affecting > 3% of children worldwide [2]. Reaching a diagnosis is a challenge, given the considerable clinical and genetic heterogeneity associated with these rare diseases [3]. Large-scale studies have identified substantial phenotypic heterogeneity and overlap between conditions, making specific genetic NDDs difficult to identify clinically. Currently, there are no biomarkers to diagnose NDDs or to differentiate between them. Rather, these disorders are categorized based on clinical presentation which is problematic since many symptoms are not unique to single NDDs, and several NDDs have clusters of symptoms in common. Similar phenotypes may be associated with different genetic mechanism. Moreover, different variants in the same gene may lead to different disease phenotypes that increase clinical heterogeneity [4, 5]. Exhaustive consultations and traditional genetic investigations are costly and often fail to arrive at a final diagnosis when no recognizable syndrome is suspected.

Next-generation sequencing (NGS) technologies have improved diagnostics of rare genetic diseases. Specifically, whole exome sequencing (WES) has led to enormous progress in deciphering monogenic forms of NDDs [2, 6]. If considering the genetic aetiology of NDDs, each of these disorders could be considered as a rare disease with overlapping clinical phenotypes. Therefore, the simultaneous analysis of several potentially mutated genes improves the diagnostic process, making it faster and more efficient. Nevertheless and despite the revolutionary advances of these approaches, in 40–60% of the families with NDDs the genetic defect remains unknown.

Current recommendations of genetic studies for diagnosis NDDs are based in the guidelines reported by Miller and collaborators reported more than 10 years ago in which chromosomal microarray (CMA) is considered the first-tier clinical diagnosis test for individuals with DD or congenital anomalies [7]. The diagnostic yield of CMA is established in approximately 15–20%. There is accumulating evidence that suggest that the diagnostic yield of whole exome sequencing in NDDs is markedly greater (30–50%) and therefore WES should be placing the first-tier clinical diagnostic test rather than CMA [8]. In the past months, it has been published the new recommendations of the American College of Medical Genetics and Genomics (ACMG) that strongly recommends applying NGS as a first- (or second-) line test in patients with congenital anomalies, DD or ID [9]. Herein, we report diagnostic yields of applying WES in 87 families with NDDs and additional data of whole genome sequencing (WGS) from 12 patients in which previous WES failed to identify a genetic aetiology. Our results support the implementation of WES as a first-tier test for NDDs.

## Material and methods

### Patients

A total of 87 families with one or several members affected by affected by NDDs were referred to the Biochemistry and Molecular Genetics department for genetic testing. This cohort reflects a heterogeneous collection of clinical presentations, making it representative of a typical genetic clinic. Before enrolment, all patients underwent an extensive diagnostic workup, including clinical evaluation and genomic profiling (fragile X syndrome, copy number variations (CNVs) in subtelomeric regions by MLPA and CMA (4 × 44 K, Agilent Technologies). The institutional review board approved the collection and use of these samples for research purposes (Ethics Committee of Hospital Clinic of Barcelona 2011/6625). Written informed consent was obtained from all subjects prior to their participation.

### Cohort structure

We used four different family series in order to test the impact of the testing strategy of an extended family analysis (more than one generation affected) *versus* a trio-based analysis or solo-based analysis (sporadic case). As shown in Table 1, 87 families were selected for WES: (i) 8 familial cases with several affected generations and many affected members. WES was performed in quartets consisting in two patients and two healthy relatives, (ii) 15 sibling couple. WES was performed in the two affected siblings with or without sequencing their parents, (iii) 26 trios sequencing the affected patient and both parents, and (iv) 38 sporadic cases in which WES was only performed to the index case. Moreover, WGS was performed in 12 patients out of the 87 families in which no pathogenic variant was identified by WES.

**Table 1:** Summary of detected variants in each family cohort

NGS strategy	Number of studies	Solved cases	% diagnosis
**WES**	83	29	35%
Large families	8	6	75%
Sibling couple	15	3	20%
Trio	26	8	31%
Sporadic cases	34	12	35%
**WGS**	12*	1	8%
**Total**	83	30	36%

^*^Cases with no pathogenic variants were identified by WES

### Sequencing and bioinformatics pipeline

Analysis of cases that underwent NGS has been previously described [10–15]. Briefly, allelic variants with MAF > 0.03 in any of the databases used (GnomAD, ExAC and 1000Genomes) and minimal coverage lower than 20% were discarded. Putative candidate variants were prioritized according to the predicted impact on coding sequence, their presence in ClinVar [16] or the Human Gene Mutation Database (HGMD) [17], zygosity and genetic mode of inheritance, clinical features and the function of the gene. The ACMG standards for interpretation of sequence variants were used to classify all reported variants [18]. Segregation analysis was performed in candidate variants by Sanger sequencing and CMA when necessary.

## Results

Our patient cohort consisted in 87 families with one or more individuals diagnosed with NDDs in which Fragile X syndrome and pathogenic CNVs were previously discarded. According to the high clinical heterogeneity of NDDs, approximately 75% (67/87) of our patients referred heterogeneous co-occurring conditions, where DD/ID was present in 90% of the patients (79/87). Other clinical co-occurring conditions included ADHD, speech disorders, epilepsy, micro/macrocephaly, and the presence of dysmorphic features/ malformations. Clinical features and genetic results of the diagnosed patients are shown in Table 2.

**Table 2:** Clinical findings and summary of pathogenic variants identified by WES or WGS

Case	Sex	Clinical findings	Gene	Transcript	Variant	Mode of inheritance and origin	OMIM	References
**Group (i)**		**Familial cases several generations**						
Patient_1	M	ID	*DYNC1H1*	NM_001376.5	c.4462dupC; p.Arg1488Profs*5	AD (mat)	ID, autosomal dominant 13 (#614,563)	
Patient_2	M	ID, microcephaly, dysmorphism	*VPS13B*	NM_017890.4	c.5998_5999delCT; p.Leu2000Alafs*2 (mat) c.10475_10476delAA; p.Lys3492Argfs*19 (pat)	AR	Cohen syndrome (#216,550)	[13]
Patient_3	M	ID	*IQSEC2*	NG_021296.1	c.3116-3_3116-2delCA	X-linked (mat)	ID, X-linked 1/78 (#309,530)	[14]
Patient_4	M	ID	*SMC1A*	NM_006306.2	c.1405C > T; p.Arg469Cys	X-linked (mat)	Cornelia de Lange syndrome 2 (#300,590)	[13]
Patient_5	M	ID	*OCRL*	NM_000276.3	c.1567G > A; p.Asp523Asn	X-linked (mat)	Dent disease (#300,555)	[13]
Patient_6	F	ID	*UBE3A*	NM_130839.4	c.2009delA; p.Asp671Metfs*3	Imprinting (mat)	Angelman syndrome (#105,830)	
**Group (ii)**		**Sibling couple**						
Patient_7	M	ID, DD, GR	*PYCR1*	NM_001282280.1	c.797 + 2delTGGG	AR	Cutis laxa type IIB (#612,940)	
Patient_8	M	Severe ID, ASD	*THOC2*	NM_001081550.1	c.3323C > T; p.Ser1108Leu	X-linked (germline mosaicism)	ID, X-linked 12/35 (#300,957)	[11]
Patient_9	M	ID, SD	*CLCN4*	NM_001830.4	c.758 G > A; p.Arg253Gln	X-linked (mat)	Raynaud-claes syndrome (#300,114)	
**Group (iii)**		**Trios**						
Patient_10	M	ASD, no speech development	*CHD9*	NM_001308319	c.3772A > C; p.Thr1258Pro	AD (de novo)		
Patient_11	F	Severe ID, DD and PDD, myoclonic seizures	*DHDDS*	NM_024887.3	c.632G > A; p.Arg211Gln	AD (de novo)	DD and seizures with or without movement abnormalities (# 617,836)	
Patient_12	M	ID, DD	*PHIP*	NM_017934.7	c. 5317 C > T; p.Arg1773Ter	AD (de novo)	DD, ID obesity, dysmorphic features (#617,991)	
Patient_13	M	Moderate ID, epilepsy, neonatal hypotonia, obesity, SD, BHD	*SETD1B*	NM_001353345.1	c. 3772 C > T; p.Arg1258Ter	AD (de novo)	ID with seizures and language delay (#619,000)	[12]
Patient_14	F	Microcephaly	*TUBB5*	NM_001293213.2	c.1201 G > A; p.Glu401Lys	AD (de novo)	Cortical dysplasia, complex, with other brain malformations 6 (#615,771)	[15]
Patient_15	M	ID	*ACSL4*	NM_022977.2	c.1030T > C; p.Ser344Pro	X-linked (mat)	ID, X-linked 63 (#300,387)	
Patient_16	M	ID, severe SD, infantile hypotonia	*THOC2*	NM_001081550.1	c.3361A > G; p.Arg1121Gly	X-linked (mat)	ID, X-linked 12/35 (#300,957)	[11]
Patient_17	M	Moderate ID, macrocephaly	*GRIA3*	NM_000828.4	c.1892G > A; p.Arg631His	X-linked (mat)	ID, X-linked 94 (#300,699)	
**Group (iv)**		**Sporadic cases**						
Patient_18	F	BHD, LD, bilateral hearing loss, cardiac malformation	*ANKRD11*	NM_013275.5	c.1940_1941delinsT; p. Arg647Leufs*6	AD (de novo)	KBG syndrome (#148,050)	
Patient_19	M	ID, BHD	*ADNP*	NM_015339.4	c.1792C > T; p.Gln598Ter	AD (de novo)	Helsmoortel-van der Aa syndrome (#615,873)	
Patient_20	M	GR, ID, compatible with Cornelia de Lange	*NIPBL*	NM_133433.4	c.385T > C; p.Ser129Pro	AD (de novo)	Cornelia de Lange syndrome 1 (#122,470)	
Patient_21	F	ID, GR, facial dysmorphism, compatible with Cornelia de Lange	*NIPBL*	NM_133433.4	c.5329-15A > G	AD (de novo)	Cornelia de Lange syndrome 1 (#122,470)	
Patient_22	F	ID, DD	*NSD1*	NM_022455.4	c.2276C > G; p.Ser759Ter	AD (de novo)	Sotos syndrome 1 (#117,550)	
Patient_23	F	PDD, macrocephaly, hypotonia	*CSPP1*	NM_024790.6	c.363_367delTAAAT; p.Leu123Rfs*19 (pat) c.2243_2244delAA; p.Glu750Gfs*30 (mat)	AR	Joubert syndrome 21 (#615,636)	
Patient_24	M	PDD	*EMC1*	NM_015047.2	c.797T > G; p.Leu266Ter (mat) c.285 T > C; p.Phe953Ser (pat)	AR	Cerebellar atrophy, visual impairment, and PDD (#616,875)	
Patient_25	F	ID, DD, microcephaly, hypotonia	*DDX3X*	NM_001356.3	c.1415A > G; p.His472Arg	X-linked (de novo)	ID, X-linked 102 (#300,958)	
Patient_26	M	Phenotype compatible with Opitz syndrome	*MID1*	NM_0000381.3	c.602_605del; p.Val201GlyfsTer11	X-linked*	Opitz GBBB syndrome, type I (#300,000)	
Patient_27	F	Dysmorphic features and LD	*PPP2R5D*	NM_006245.3	c.592G > A; p.Glu198Lys	AD (de novo)	Mental retardation, autosomal dominant 35 (#616,355)	
Patient_28	M	ASD, ID	*WAC*	NM_016628.5	c.1280_1281delCTinsGAG	AD (de novo)	Desanto-Shinawi syndrome (#616,708)	
Patient_29	M	ID and dysmorphic features	*KCNT2*	NM_198503.5	c.569G > A; p.Arg190His	AD (de novo)	Developmental and epileptic encephalopathy 57 (#617,771)	[20, 21]
Patient_30	F	ID, absent speech, dysmorphic features	*CNOT3*	*NM_014516.3*	c.169C > T (p.Arg57Trp)	AD (de novo)	Intellectual developmental disorder with speech delay, autism, and dysmorphic facies	
**Group (v)**		**Whole genome sequencing**						
Patient_31	M	Severe ID, absent speech, BHD	*TRAPPC9*	NM_031466.7	c.1037G > A; p.Gly346Glu (mat) arr8q24(141382973_141473138) × 1 (pat)	AR	ID, autosomal recessive 13 (#613,192)	[10]

ID: intellectual disability; DD: developmental delay; GR: growth retardation; ASD: autistic spectrum disorder; SD: speech delay; BHD: behavioural disorder; PDD: psychomotor development delay; LD: learning disabilities; *mother not available

The use of NGS technologies allowed identifying the causative genetic alteration in 36% of the cases, which is a significant increase over conventional CMA testing. The initial cohort included 97 cases, with a diagnostic value of 8.2% and 32% for CMA and NGS, respectively. Using NGS we achieved genetic diagnosis in 31 families identifying all patterns of inheritance (Table 3). Variants of unknown significance (VUS) have not been included in this report.

**Table 3:** Diagnostic yield by WES and WGS in the analyzed cohorts

Total positive diagnosis	Cases (n = 31)
Autosomal dominant	15 (48.4%)
De novo	14
Inherited	1
X-linked	10 (32.3%)
Dominant	1
Recessive	9
Autosomal recessive	5 (16.1%)
Homozygous	1
Compound heterozygous	4
Imprinting	1 (3.2%)

**Fig. 1:**
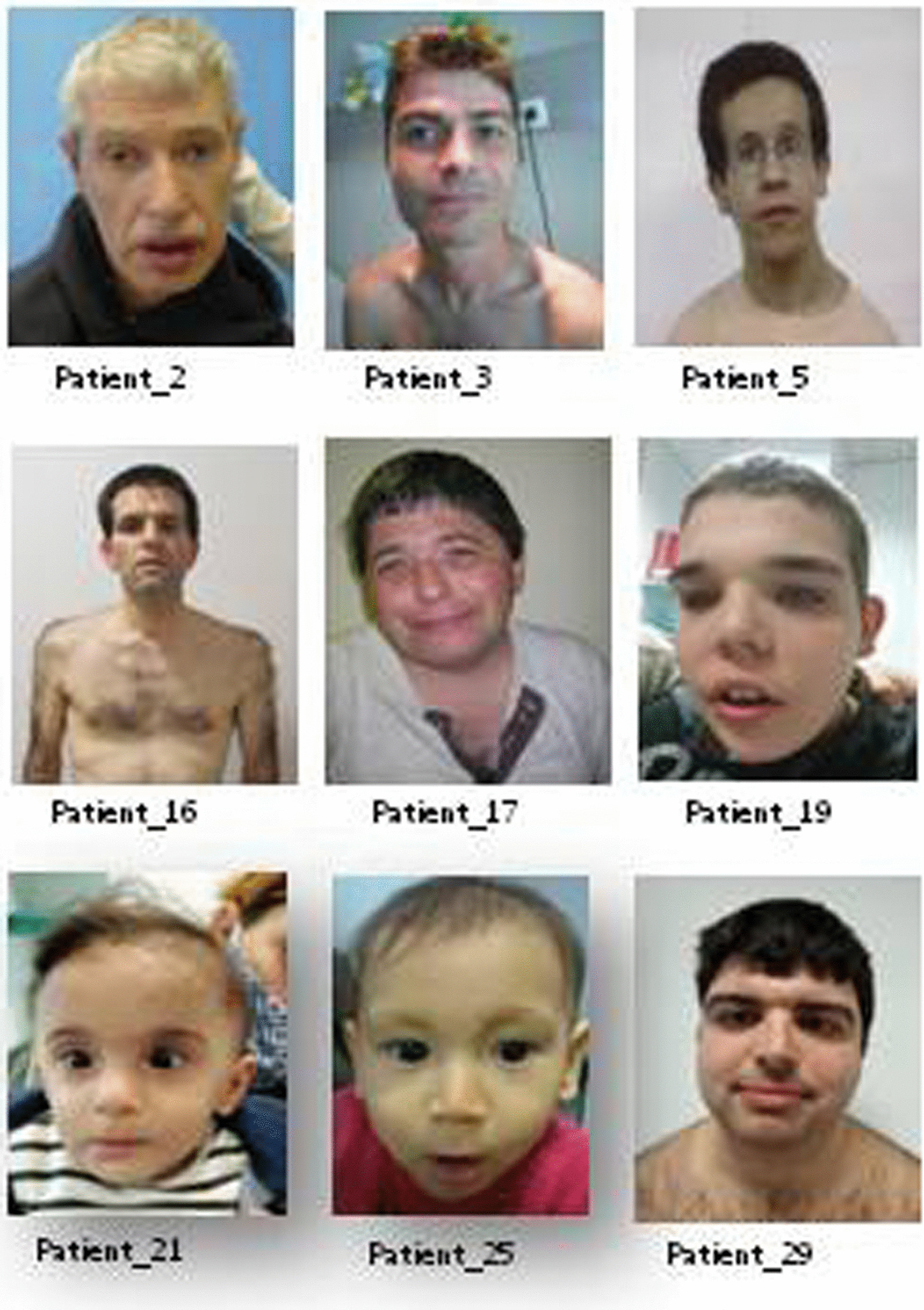
Clinical pictures of 9 of the patients with genetic diagnosis identified in this study. Clinical manifestations are summarised in Table 2

In Fig. 1 are shown clinical features of some of the patients with definitive diagnosis. WES identified causative alterations in 30 families out of 87. Diagnostic yields ranged from 20 to 75% depending on the cohort structure (Table 1). Statistically analysis identified significant differences between the group of large families, with several generations affected, and the rest of the groups (p < 0.001). While the diagnostic yield in the large families was 75%, the other achieved similar rates of pathogenic variant detection (sibling couple 20%; 31% trio-based; and 34% in sporadic cases). Among the 57 families that remained undiagnosed, WGS was applied to 12 patients and causative variants were only found in one (8%) of the families corresponding to a couple of affected siblings (Tables 1, 2).

### Autosomal dominant inheritance

The most common mode of inheritance identified in our cohort was AD which was found in approximately half of the cases (Table 3). Among them, 14 out of the 15 cases were found to be caused by de novo mutations and the remaining case (patient 1) was found to carry a loss-of-function (LOF) variant in *DYNC1H1* gene inherited from his affected mother (Tables 2, 3).

Most of the de novo pathogenic variants were found to be novel and detected in different genes previously associated with NDDs (*ADNP*, *ANKRD11*, *CNOT3*, *DHDDS*, *NSD1*, *PHIP*, *PPP2R5D*, *SETD1B*, *TUBB5* and *WAC*). We also found three patients with previously reported variants, two of them with de novo variants in *NIPBL* gene and one with a de novo variant in the *KCNT2* gene (Table 2). Patient 20 was found to carry the missense variant c.385T > C (p.Ser129Pro) in *NIPBL* present in gnomAD database with an extremely low allelic frequency (rs141976717); patient 21 carried the splicing variant c.5329-15A > G in *NIPBL* reported in the literature to result the skipping of exon 28 without altering the reading frame [19]; and patient 29 carried the missense variant c.569G > A (p.Arg190His) in the *KCNT2* gene previously reported in the literature [20, 22].

On the other hand, we identified a de novo missense variant in the *CHD9* gene (patient 10), which is currently not associated with ID but some patients with autistic features have been already reported in HGMD database.

### X-linked inheritance

Our results have identified 10 families with pathogenic variants in 9 genes located in the X-chromosome (*ACSL4, CLCN4, DDX3X, GRIA3, IQSEC2, MID1, OCRL, SMC1A* and *THOC2*), representing 32% of the solved cases. We identified two families (patient 8 and patient 16) with pathogenic variants in the *THOC2* recently associated with the Mental retardation, X-linked 12/35 syndrome (OMIM # 300,957).

All pathogenic XL variants, except by one, were detected in affected male patients and were associated with XL-recessive pattern of inheritance (Tables 2, 3). Segregation analysis demonstrated that pathogenic variants were inherited from asymptomatic mothers. However, segregation analysis was not available in patient 26 who was found to carry a hemizygous frameshift variant in *MID1* gene (Table 2).

On the other hand, one female (patient 25) was found to carry a de novo missense pathogenic variant in *DDX3X* gene, associated with and ID disorder with XL-dominant pattern of inheritance.

### Autosomal recessive inheritance

In our cohort, 16% (5/31) of the families were found to carry genetic alterations associated with an AR pattern of inheritance (Table 3). WES identified three families with compound heterozygous variants in AR associated genes, namely, *VPS13B* (patient 2)*, CSPP* (patient 23) and *EMC1* (patient 24) and one consanguineous family with a homozygous variant in the *PYCR1* (patient 7) gene (Table 2). In addition, WGS allowed the identification of an structural variant (SV) and a missense variant in compound heterozygosis in the *TRAPPC9* (patient 31) gene (Table 2).

### Imprinting inheritance

Finally, mutations in imprinted genes represented 3% of our diagnostic cohort (Table 3). Only one case (patient 6) was found to carry a LOF variant in the imprinted *UBE3A* gene inherited from her asymptomatic mother.

## Discussion

The past decade has seen a rapid development of advancements in genetics and genomics, allowing an unprecedented identification of mutations that are involved in complex neurodevelopmental conditions. In the last years, WES has emerged as a comprehensive and cost-effective approach for discovering pathogenic variants in rare monogenic diseases.

In this report we present pathogenic variants detected by WES in a cohort of 87 families with NDDs. The resulting diagnostic yield in the sequenced cohort was 34%. After considering testing strategy based on family series, our analysis determined that extended families approach had a positive impact on diagnostic yield. However, the most common type of family that arrives to a medical genetics consultation for NDDs counselling is based on an isolated cases or a couple of siblings within a family, representing in our cohort more than 70% (64/87) of the patients. On the other hand, no differences were found in the diagnosis rate between the other cohort structures (siblings, trios and sporadic cases). Nevertheless, trio-based analysis reduced the turnaround time of genetic diagnosis (data not shown) and despite not having WES data from parents, segregation analysis were in most cases essential in order to definitively classify variants as disease causing.

De novo variation has been proposed as a major pathomechanism in NDDs representing approximately 20–30% of ASD and ID cases [22, 23]. Our results identified monogenic aetiology due to de novo mutation in 48.4% (15/31) of the patients with genetic diagnosis, 14 of them in autosomal genes and 1 in an X-linked gene, representing the main source of genetic alteration in our cohort. Besides de novo variants, XL recessive disorders were found in 29% of the diagnosed patients (9/31), highlighting the role of the X-chromosome in underlying genetic basis of NDDs in affected males. To date, there are more than 140 genes on the X-chromosome related to cognition associated with ID [24]) and thus, variants in the X chromosome greatly contribute to ID in males.

Regardless the pattern of inheritance, most of the identified genes are implied in syndromic neurodevelopmental disorders, such as Cornelia de Lange syndrome (CdLS), Dent disease or Cohen syndrome, which are considered clinically recognizable syndromes. Nevertheless, several of the identified variants are rare and in some cases the clinical manifestations do not correspond to the classic syndromic forms or clinical features are not really specific. For example, patient 29 was referred to clinical consultation for presenting ID and dysmorphic features. Results from NGS identified a de novo previously reported variant in the *KCNT2* gene, usually associated with Developmental and epileptic encephalopathy type 57 (OMIM # 617771). This syndrome is characterized by epileptic encephalopathy, global DD with hypotonia, variably impaired intellectual development and poor or absent language. The *KCNT2*-associated developmental and epileptic encephalopathy comprises West syndrome, Lennox-Gastaut syndrome as well as epilepsy of infancy with migrating focal seizures. Currently, there are 4 patients with de novo mutations in the Arg190 residue described in the literature [20, 21]). Our patient was 3 years old and presented with development and speech delay, bruxism, attention deficit, repetitive behaviors and physical contact avoidance. He presented some facial dysmorphisms such as elongated face with a broad-based nose, a short filter and little marked slight prognathism. He had blonde hair color, unlike her parents and siblings who had brown hair color. Curiously there were no epilepsy attacks. In consonance with our results, the patients recently reported by Jackson and collaborators (2021) present a recognizable neurodevelopmental disorder without epilepsy [21]). All patients described up to date with variants affecting p.Arg190 of the *KCNT2* gene present ID, neonatal hypotonia, hirsutism, thick hair, prominent eyebrows, long eyelashes, and diastema [21].

NGS is also unveiling a large number of VUS, which effectively represent mutations where the pathogenicity and the function of the gene involved is unclear, hampering their endorsement to a clinical phenotype. The process of variant prioritization is performed based on several factors, including their possible presence in databases such as gnomAD, ClinVar and HGMD; the relevance to the reported clinical features, the impact of the variant or the associated mode of inheritance, among others. In order to determine whether a VUS may be associated with a patients’ phenotype, additional studies are needed including segregation analysis or functional studies. There are several possibilities of functional tests that can be performed to answer questions about the function of genes and the functional consequences of genetic variants. RNA studies are the easiest and fastest ways to determine if a variant of unknown significance could be pathogenic. Nevertheless it is only indicated in variants that produce changes in mRNA expression levels, for example, variants that cause alternative splicing events or that cause loss of function. Protein expression studies have already proven their value to demonstrate pathogenicity of genetic variants. Another strategy is to test the genetic variants in model systems (for example cell culture models or animal models) [reviewed in 25.

**Fig. 2:**
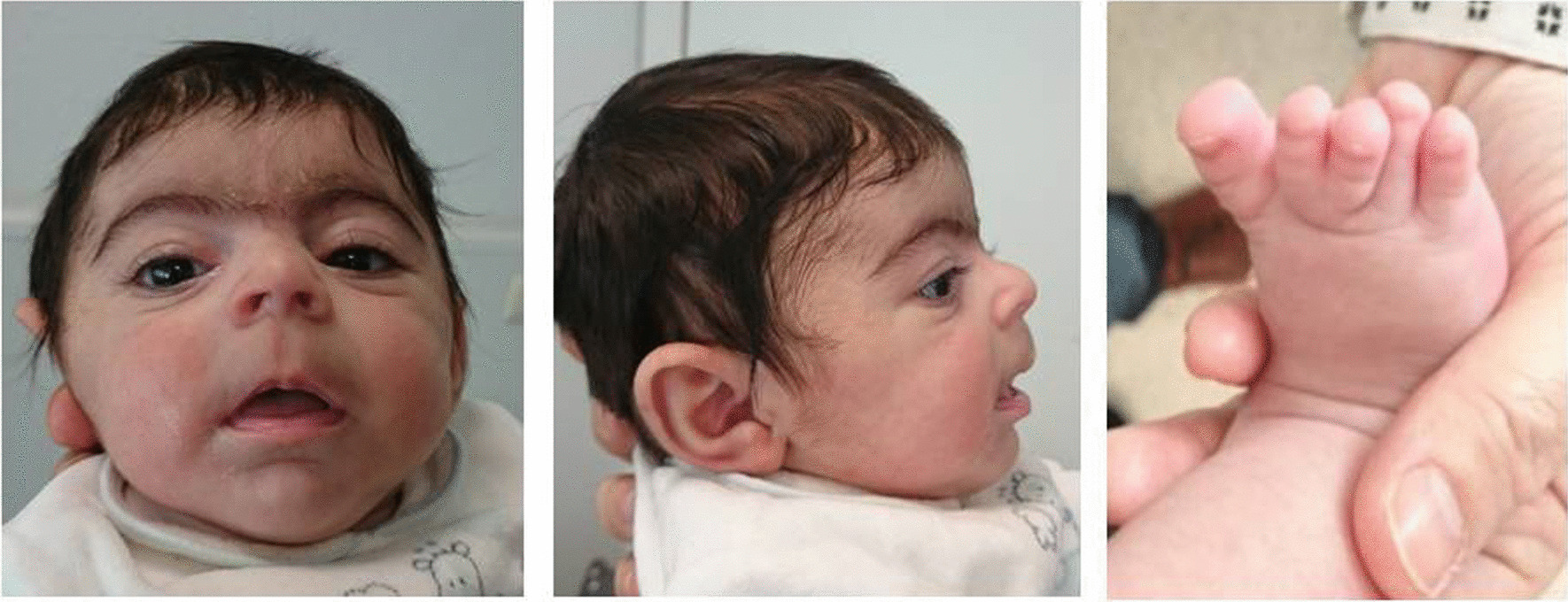
Clinical picture of patient 20 carrier of a de novo variant in *NIPBL* gene (NM_133433.4: c.385T > C; p.Ser129Pro)

Another approach is the reinterpretation of VUS in a given period later from initial analysis, which has been proven to increase diagnostic efficacy [26]. In our cohort, we identified an ultra-rare missense variant in the *NIPBL* gene (c.385T > C; p.Ser129Pro; rs141976717) in case 20 clinically diagnosed with CdLS. This patient has global cognitive and growth retardation, microcephaly, and dysmorphia. Physical examination revealed obvious clinical signs of CdLs such as long philtrum, long eyelashes, high-arched palate and anteverted nares, hypospadias (Fig. 2). Other clinical manifestations were the absence of language, mild hypertonia of the lower and upper extremities, and syndactyly of the 2nd and 3rd toes. Initially, this patient underwent to an external laboratory to perform a CdLS gene panel focused on the five current genes associated to this syndrome. A missense c.385T > C variant in a non-conserved position was reported. In silico tools indicated benign computational verdict based on 10 benign versus 2 pathogenic predictions and it was described in gnomAD database with an ultra low allelic frequency (0.004%). On the basis of these observations, this variant was initially discarded to be responsible of the patient’s phenotype. WES was later performed in this patient and no additional genetic alterations were identified associated to NDDs. After reanalysis this variant, segregation analysis was performed reveling that the variant c.385T > C was originated de novo in the patient. Although we cannot rule out the presence of an intronic variant in any of the CdL genes, taking into account that the majority of CdLS patients have de novo heterozygous missense variant in *NIPBL*, this variant was reclassified as likely pathogenic.

Despite the significant improvement in diagnostics of rare diseases using WES, a significant proportion of cases with a likely genetic aetiology remain undiagnosed. A step further WES, is WGS which allows the identification of sequence variants in non-coding regions making up the vast majority of human DNA [27]). However, there is evidence that the increased rate of WGS compared to WES is due to its efficiency for detecting potential disease-causing mutations within single nucleotide variants (SNV) in regions not properly covered by WES or SV not detected by this technology [28]). In our cohort, WGS was applied to 12 out of the 57 patients which remain undiagnosed, identifying only causative variants in 1 of the patients (Table 1). This patient (patient 31) was found to carry a missense variant and an intragenic deletion of 90 Kb, not detected neither by CMA nor WES [10].

The application of NGS technologies and the development of several web-based tools such as GeneMatcher (http://www.genematcher.org) to share phenotype and genotype data to broaden the exchange between scientific and medical teams have allowed the discovery of new genes responsible for the disease. In our cohort, we identified two families diagnosed with pathogenic variants in *THOC2* gene (Table 2), who were previously reported as a series of cases describing the phenotype associated with THOC-related syndrome [11]. In addition, GeneMatcher tool allowed the delineation of a genome-wide DNA methylation signature for SETD1B-related syndrome, in which the patient herein reported (patient 13) was also included [12]. Interestingly, one of the patients of our cohort was found to carry a de novo likely pathogenic variant in the *CHD9* gene, which is a strong genetic candidate for NDDs that not yet been associated with ID. The CHD (Chromodomain Helicase DNA-binding protein) gene family consists of nine genes (*CHD1*–*CHD9*), and pathogenic variants in all CHD genes except *CHD9* are implicated in NDDs [29]. Recently, other groups have suggested a possible implication of *CHD9* variants in ASD patients [30–32]. Given the role of this gene as a transcriptional regulator and chromatin decompressor and the involvement of the rest of the CHD family genes in several NDDs, we consider that *CHD9* is a candidate gene for ASD.

## Conclusions

Currently, there are more than 2000 genes associated with NDDs making the implementation of NGS in the diagnostic flowchart of NDDs essential. Our results support the use of NGS for genetic diagnosis of NDDs as a first-tier test since it has a clear higher diagnostic rate compared to CMA (33% vs 10%). However, it should be taken into consideration that pathogenic CNVs were discarded by CMA in all patients presented in this report before performing NGS studies which might be a limitation of the study. On the other hand, new bioinformatic pipelines allow the detection not only of SNV but also reliable CNVs through WES data which further reinforces this change in diagnostic algorithm. The genetic architecture of NDDs is complex and interdisciplinary approaches combining genetics, functional genomics and biological models will be essential.

## Data Availability

Please contact corresponding author for data requests.

## Abbreviations

ACMG: American College of Medical Genetics and Genomics
ADHD: Attention deficit hyperactivity disorder
ASD: Autism spectrum disorder
CdLS: Cornelia de Lange syndrome
CGH: Comparative genomic hybridization
CHD: Chromodomain Helicase DNA-binding
CMA: Chromosomal microarray
CNV: Copy number variant
DD: Developmental delay
HGMD: Human Gene Mutation Database
GR: Growth retardation
ID: Intellectual disability
LOF: Loss-of-function
MLPA: Multiplex Ligation-dependent Probe Amplification
NDDs: Neurodevelopmental disorders
NGS: Next-generation sequencing
OMIM: Online Mendelian Inheritance in Man
SNV: Single nucleotide variant
SV: Structural variant
VUS: Variant of unknown significance
WES: Whole exome sequencing
WGS: Whole genome sequencing
